# N-Rich Doped Anatase TiO_2_ with Smart Defect Engineering as Efficient Photocatalysts for Acetaldehyde Degradation

**DOI:** 10.3390/nano12091564

**Published:** 2022-05-05

**Authors:** Mingzhuo Wei, Zhijun Li, Peijiao Chen, Lei Sun, Shilin Kang, Tianwei Dou, Yang Qu, Liqiang Jing

**Affiliations:** Key Laboratory of Functional Inorganic Materials Chemistry (Ministry of Education), School of Chemistry and Materials Science, International Joint Research Center for Catalytic Technology, Heilongjiang University, Harbin 150080, China; mingzhuo0828@163.com (M.W.); chenpeijiao0@163.com (P.C.); 2021022@hlju.edu.cn (L.S.); 18846423501@163.com (S.K.); doutw1992@126.com (T.D.)

**Keywords:** anatase TiO_2_, N-rich doping, defect healing, charge separation, photocatalytic acetaldehyde degradation

## Abstract

Nitrogen (N) doping is an effective strategy for improving the solar-driven photocatalytic performance of anatase TiO_2_, but controllable methods for nitrogen-rich doping and associated defect engineering are highly desired. In this work, N-rich doped anatase TiO_2_ nanoparticles (4.2 at%) were successfully prepared via high-temperature nitridation based on thermally stable H_3_PO_4_-modified TiO_2_. Subsequently, the associated deep-energy-level defects such as oxygen vacancies and Ti^3+^ were successfully healed by smart photo-Fenton oxidation treatment. Under visible-light irradiation, the healed N-doped TiO_2_ exhibited a ~2-times higher activity of gas-phase acetaldehyde degradation than the non-treated one and even better than standard P25 TiO_2_ under UV-visible-light irradiation. The exceptional performance is attributed to the extended spectral response range from N-rich doping, the enhanced charge separation from hole capturing by N-doped species, and the healed defect levels with the proper thermodynamic ability for facilitating O_2_ reduction, depending on the results of ∙O_2_^−^ radicals and defect measurement by electron spin resonance, X-ray photoelectron spectroscopy, atmosphere-controlled surface photovoltage spectra, etc. This work provides an easy and efficient strategy for the preparation of high-performance solar-driven TiO_2_ photocatalysts.

## 1. Introduction

Wide bandgap oxide photocatalysts (such as classical TiO_2_) have displayed all-round activities because of the adequate potentials of the conduction and valence bands for redox [1,2,3,4,5,6]. However, their low solar-spectrum utilization (<5%), one of the most critical bottlenecks for practical application, has been identified [7]. Because O 2*p* primarily contributes to the valance band of a wide bandgap oxide, it is feasible to narrow the bandgap by elevating the valence band by doping anions with higher potential energy to increase light utilization [8,9,10]. Since oxygen and nitrogen have similar properties such as polarizability, electronegativity, and ionic radii, nitrogen (N) doping is perfectible in this aspect [11,12]. Meanwhile, N with long-pair electrons facilitates trapping holes for enhancing charge separation [13]. To date, extensive N doping techniques for improving photoactivities have been established. Most studies, on the other hand, obtained low-concentration N doping results, which are mainly attributed to the high metal–oxygen bonding energy (for example, Ti–O = 346.4 kJ/mol) and imperfect methodologies [14,15,16]. The development of efficient strategies for preparing high-efficiency N-rich doping oxides is still a work in progress.

Among the developed strategies, nitridation in an ammonia atmosphere has been demonstrated to be the most efficient method for N doping [17]. To overcome the barrier of breaking the metal–oxide bonds, a high temperature is required for N-rich doping in general. The phase transformation of oxides, however, normally occurs at a high temperature. For example, more than 600 °C is required to prepare the N-rich doping anatase TiO_2_, but such a high temperature will cause phase transformation from the high-active anatase to the inferior-rutile one [18,19]. As a result, the thermal stability of anatase TiO_2_ is crucial for N-rich doping at high temperatures. Fortunately, the strategy of phosphoric acid modification was previously developed to improve the thermal stability of anatase TiO_2_ as high as 750 °C, making phosphate-modified anatase TiO_2_ (PTO) an available substrate to prepare N-rich doped TiO_2_ at a high temperature [20].

The charge difference between lattice O^2−^ and dopant N^3−^ in N-TiO_2_ inevitably introduces some associated defects, such as oxygen vacancies (O_v_) and Ti^3+^ defects [21,22]. O_v_ at shallow energy levels are usually demonstrated to trap the photogenerated electrons, which enhance charge separation and thermodynamically favor O_2_ activation [23,24]. O_v_ or the relative Ti^3+^ at deep energy levels from a high N doping concentration can be either advantageous or disadvantageous, depending on the situation. In some cases, O_v_ at deep states are intentionally pursued in cases of self-doped TiO_2,_ when the optical absorption at wavelengths is longer than 400 nm [25,26]. However, they can also have a net disadvantageous effect when they are not exploited by optical absorption, so that their overall effect as recombination centers is to hamper the charge mobility and thermodynamically reduce O_2_, resulting in decreased photocatalytic activity [27,28]. Usually, a high temperature promotes N-rich doping, but it also produces numerous O_v_ and Ti^3+^ defects at deep energy levels. Liu’s group recently prepared a boron-doped TiO_2_ as a precursor to achieve an N-rich doping by using boron ions to balance the charge difference so as to avoid Ti^3+^ at deep energy levels [29]. However, TiB_2_ as a raw material is unusual for a versatile application. Developing a controllable method for healing the adverse O_v_ at deep energy levels for N-rich doped TiO_2_ is of great significance to the high photocatalytic activity. As for the essence of O_v_ at deep energy levels, Ti^3+^ defects are typical and important species. Oxidizing Ti^3+^ defects into Ti^4+^ is feasible to heal O_v_ at deep energy levels. It has been reported that Ti^3+^ is an active site to achieve a photo-Fenton-treated reaction, resulting in the oxidation of Ti^3+^ defects into Ti^4+^ [30]. This interesting reaction is of great importance and opens our minds to controllably heal O_v_ without introducing impurities. To date, there is no work referring to the utilization of the photo-Fenton-treated reaction to control the vacancies in N-TiO_2_.

In this work, N-rich doped anatase TiO_2_ nanoparticles (4.2 at%) were successfully fabricated via high-temperature nitridation based on thermally stable H_3_PO_4_-modified TiO_2_. Subsequently, the associated deep-energy-level defects such as O_v_ and Ti^3+^ were successfully healed by a smart photo-Fenton-treated oxidation strategy. Under visible-light irradiation, the healed N-doped TiO_2_ exhibited a ~2-times higher activity of gas-phase acetaldehyde degradation than the non-treated one, and even better than the standard P25 TiO_2_ under UV-visible-light irradiation. The high activity was attributed to the extended light response due to N-rich doping, the enhanced charge separation from hole capturing by N-doped species, and remaining O_v_ after healing of deep-energy-evel defects with a proper thermodynamic ability for facilitating O_2_ reduction. This work uncovers the N-rich doping in TiO_2_-photocatalyst-produced defects at various energy levels and their influence on charge separation and O_2_ activation, as well as an efficient technique for engineering the defects for high-performance solar-driven TiO_2_ photocatalysts.

## 2. Materials and Methods

### 2.1. Synthesis of H_3_PO_4_-Modified Anatase TiO_2_ Nanoparticles

Anatase TiO_2_ nanoparticles were prepared by a common sol–hydrothermal method [20]. The typical procedures are described in the Supporting Information. For H_3_PO_4_ modification, 0.5 g of as-prepared anatase TiO_2_ nanoparticles was dispersed in 30 mL of H_3_PO_4_ solution (0.1 M) under continuous stirring for 4 h. The precipitate was then centrifuged and washed once with absolute ethanol before being dried at 80 °C for 10 h and calcined at 450 °C for 1 h to yield H_3_PO_4_-modified anatase nanoparticles, denoted as PTO.

### 2.2. Synthesis of Nitrogen-Doped PTO

Nitrogen-doped PTO was prepared by heating PTO with pure NH_3_. In a typical experiment, 0.2 g of PTO was put into a quartz boat in a tubular furnace, and then, the temperature was elevated to 500–650 °C in steps of 5 °C/min in a NH_3_ flow (50 mL/min). PTO was treated with NH_3_ for 2 h before naturally cooling down to room temperature. The samples are denoted as NPTO-T, where -T represents the treatment temperature in ammonia. As a reference sample, NTO-500 was prepared by the treatment of TO with a pure NH_3_ atmosphere at 500 °C.

### 2.3. Defects Healing of NPTO

Photo-Fenton-treated healing process: 0.2 g of NPTO-650 was placed in 30 mL of 30% H_2_O_2_ solution and stirred under 300 W Xenon-light irradiation for 1–3 h at room temperature. The sample is denoted as R_L-H_2_O_2__-t-NPTO-650, where -t represents the treatment time. R_H_2_O_2__-t-NPTO-650 as reference samples were prepared by the same treatment process, except under dark condition.

### 2.4. Oxidation Healing Process

There was 0.2 g of NPTO-650 put into a quartz boat in a tubular furnace, and then, the temperature was elevated to 300–450 °C in steps of 5 °C/min in an O_2_ flow (50 mL/min). NPTO-650 was treated with O_2_ for 0.5–1.5 h before cooling down to room temperature naturally. The samples are denoted as R_O_2__-t-T-NPTO-650, where -T- and -t represent the treatment temperature and time, respectively.

### 2.5. Photocatalytic Activity Evaluation

The photocatalytic activity was evaluated by gas-phase acetaldehyde degradation. In a typical process, 0.1 g of catalyst was uniformly coated on the surface of a 5 cm × 5 cm glass sheet, which was placed in a self-made stainless steel cell (Volume: 1 L) with a quartz window. Then, a gas mixture (800 ppm acetaldehyde, 20% O_2_, and about 80% N_2_) was continuously passed through the reactor for 2 h, reaching adsorption equilibrium in the dark. The acetaldehyde concentration was determined with a gas chromatograph (Shanghai Kechuang, GC-2002, Shanghai, China) equipped with a flame ionization detector after reaction under different light irradiation (visible light: 150 W high-pressure Xenon lamp with a 420 nm cutoff filter; the incident integrated irradiance of the lamp on the sample surface was 58 mW/cm^2^; single-wavelength light: 100W LED light with different wavelengths; the incident integrated irradiance of the lamp on the sample surface was 20 mW/cm^2^). The power spectrum vs. wavelength of the Xe lamp and LED lamp can be seen in Figure S1.The cycle test was performed in the same way, and the reactor and photocatalyst were purged by pure N_2_ before the repeated experiment.

### 2.6. Characterizations

The powder X-ray diffraction (XRD) analyses were recorded with a Bruker D8 Advance diffractometer (CuKα radiation, Billerica, MA, USA). The morphologies and element distribution of the photocatalysts were obtained by a scanning electron microscope (SEM, SIGMA-500, ZEISS, Jena, Germany) and transmission electron microscope (TEM, JEM-F200, JEOL, Showa, Tokyo, Japan) with energy dispersive X-ray spectroscopy (EDS). The UV-visible diffuse reflectance spectra (DRS) of the photocatalysts were investigated in the range of 220–800 nm by the Shimadzu UV-2700 model spectrophotometer (Suzhou, China). Fourier transform infrared (FT-IR) spectroscopy was performed using a Thermo Scientific Nicolet iS50 FT-IR spectrometer (Waltham, MA, USA). The Raman spectra were measured with a Renishaw inVia Confocal Raman spectrometer (Loucestershire, London, UK). X-ray photoelectron spectra (XPS) surveys were tested on a Kratos-AXIS ULTRA DLD (Manchester, UK), Aluminum (Mono) spectrometer. The electron spin resonance (ESR) and DMPO spin-trapping ESR analyses were carried out on a Bruker EMXPLUS model spectrometer (Billerica, MA, USA) at ambient temperature. The surface photovoltage spectra (SPS) were implemented on home-built equipment with a lock-in amplifier (SR830, Silicon Valley, CA, USA) synchronized with a light chopper (SR540, Silicon Valley, CA, USA).

## 3. Results

### 3.1. N-Rich Doping

Anatase TiO_2_ (TO) and H_3_PO_4_-modified TiO_2_ (PTO) were synthesized by using a common sol–hydrothermal method as described in our previous work [20]. The XRD patterns (Figure S2a), SEM images (Figure S2b), and DRS (Figure S2c) illustrate that there was no change in the crystal phase (anatase), particle sizes (5~10 nm), and bandgap (3.2 eV) of TO before and after phosphoric acid modification. FT-IR spectra (Figure S2d) showed a new absorption peak at 1010~1250 cm^−1^ in PTO, which can be attributed to PO_4_^3−^. As shown in Figure S1e, XPS confirmed the phosphate from the P 2*p* peak at 133.6 eV [31,32]. In addition, the SPS response of PTO in Figure S1f shows a higher signal than TO, indicating that H_3_PO_4_ modification can improve the charge separation of TO. This is because the appropriate modified PO_4_^3−^ can promote the oxygen adsorption and subsequently capture photogenerated electrons [33]. Based on the XRD patterns (Figure S3a,b), the phase transformation from anatase to rutile appeared in TO after thermal treatment at 550 °C and was completely converted into the rutile phase until 650 °C. Compared with TO, PTO retained a pure anatase phase after thermal treatment at 650 °C, displaying high thermal stability. This was further confirmed by the DRS. The absorption edge of TO exhibited a red shift after 550 °C treatment due to the formation of rutile TiO_2_ (Figure S2c), but no change of PTO could be detected even after 650 °C. PTO with high thermal stability provides the feasibility of N-rich doped anatase TiO_2_.

**Figure 1:**
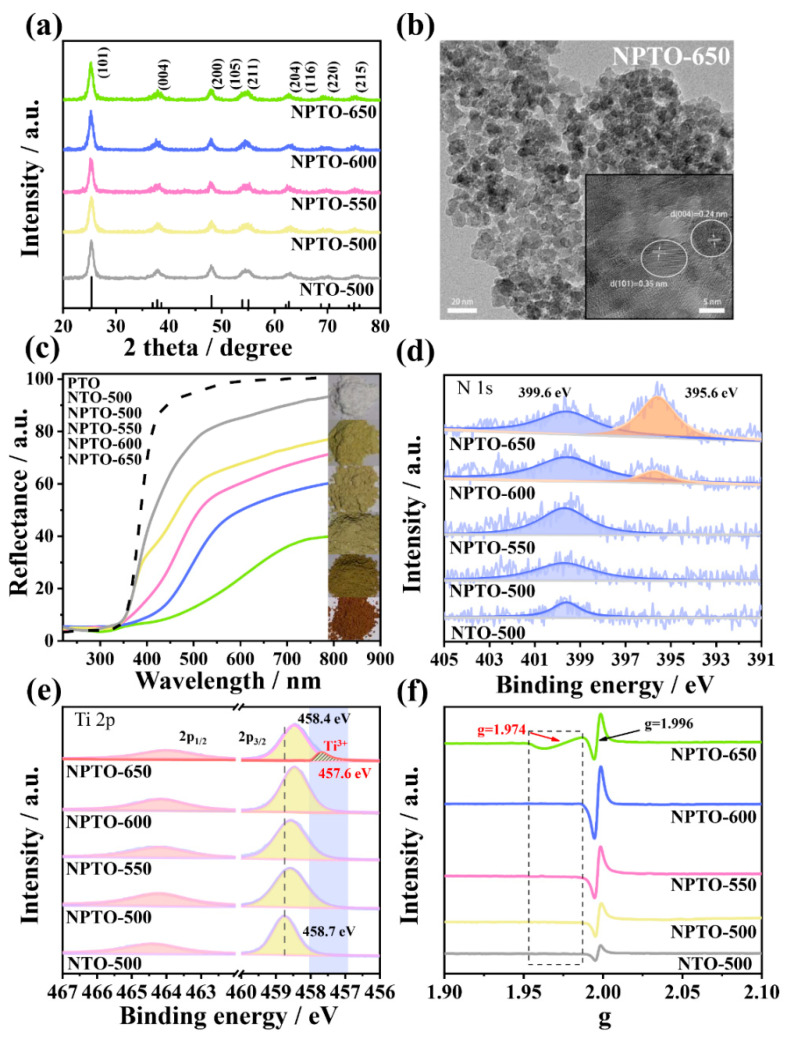
(**a**) XRD patterns of NTO-500 and NPTO-T (T: temperature of nitridation). (**b**) TEM and HR-TEM images of NPTO-650. (**c**) DRS of PTO, NTO-500, and NPTO-T. XPS for N 1*s* (**d**) and Ti 2*p* (**e**) of NTO-500 and NPTO-T. (**f**) ESR spectra of NTO-500 and NPTO-T.

Subsequently, the effects of temperature on the as-prepared N-doped PTO (NPTO-T) were investigated. The XRD patterns of NPTO-T and NTO-500 in Figure 1a reveal that both phases of NTO-500 and NPTO-T were anatase. TEM image (Figure 1b) illustrated the particle size of NPTO-650 to be approximately 5–10 nm. The anatase phase was further confirmed by the HR-TEM image (inset Figure 1b) according to the interplanar distances of (004) and (101) planes of anatase TiO_2_ [34]. Moreover, the morphology of the as-prepared samples was characterized by SEM images (Figure S4). There was no obvious difference between NTO-500 and NPTO-T. NTO-650 exhibited an obvious change in the dramatically increased particle size because of the growth of grains during the crystal-phase transition. The DRS in Figure 1c shows that NTO-500 only exhibited a weak shoulder-like visible-light absorption because of the comparatively low content of doped N at 500 °C. However, the light absorption of NPTO-T obviously enhanced with the increasing nitridation temperature. The bandgap edge of NPTO-650 surprisingly shifted to 700 nm. The corresponding photos in Figure 1c display the color change of samples from white (PTO) to pale yellow (NPTO-500), and finally, dark red (NPTO-650).

The N-doped species in the TiO_2_ lattice were further studied by XPS. In the N 1*s* spectra (Figure 1d), all the samples presented a peak at 399.6 eV, which was ascribed to the interstitial N-like Ti–O–N [35]. The intensity increased along with the rising temperature, indicating the increased N content. Noticeably, an additional peak appeared at 395.6 eV in NPTO-600 and NPTO-650, which was ascribed to the substituted N for lattice oxygen by forming O–Ti–N bonds [36]. The content of substituted N in NPTO-650 was higher than that in NPTO-600. In addition, the XPS valence band spectra (Figure S5a) showed that the valence band position of PTO was 3.0 eV, while those of NPTO-600 and NPTO-650 were 2.2 eV and 1.7 eV, respectively. The elevated valence band extends the visible-light response of TiO_2_.

The chemical state of Ti in different N-doped TiO_2_ was studied by XPS and electron spin resonance (ESR). In Figure 1e, NTO-500 has two peaks at 464.4 and 458.7 eV, respectively, which correspond to the typical Ti^4+^–O bond [37]. In comparison to NTO-500, there was a noticeable red-shift of Ti 2*p* in NPTO-T samples, which gradually improved with increasing temperature. NPTO-650 exhibited a new binding energy peak at 457.6 eV, which was ascribed to the Ti^3+^ species [38]. By analyzing the previous literature [29,39], ESR (Figure 1f) consistently showed sharp signals at g = 1.996 in all samples, implying the defects associated with the N doping amount. The additional weak signal at g = 1.974 was attributed to the Ti^3+^ defects in NPTO-650, which coincided with the XPS [29].

**Figure 2:**
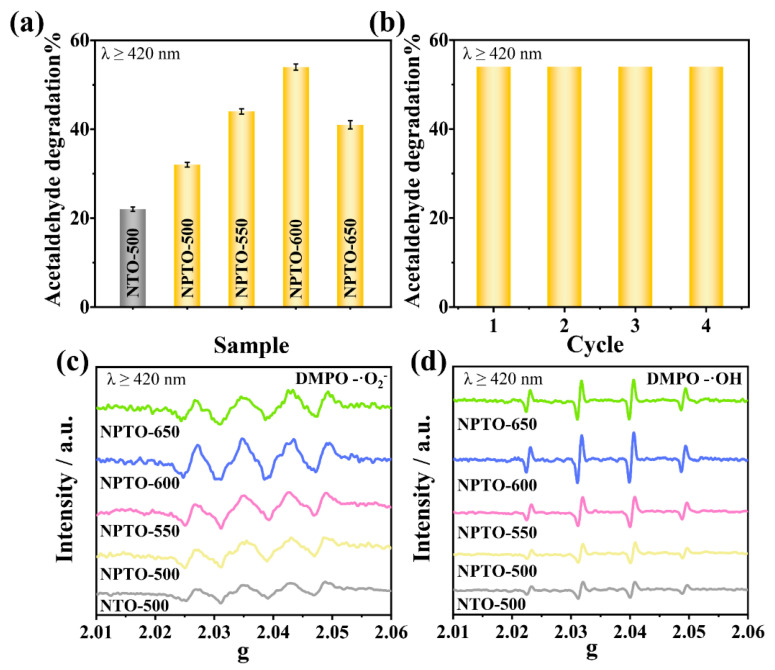
(**a**) Photocatalytic activities of NTO-500 and NPTO-T for acetaldehyde degradation under visible-light irradiation for 1 h. (**b**) Photocatalytic cycling tests of NPTO-600 under visible-light irradiation. DMPO spin-trapping ESR spectra of produced superoxide radicals (**c**) and hydroxyl radicals (**d**) over NTO-500 and NPTO-T samples under visible-light irradiation.

To clarify the effect of N doping and the associated defects on the photocatalytic performance, the photocatalytic activities of the as-prepared N-doped TiO_2_ were tested for acetaldehyde degradation under visible-light irradiation (Figure 2a). All of the NPTO-T samples exhibited higher activities than NTO-500, while NPTO-600 showed the best photocatalytic performance. The degradation rate of NPTO-600 with good stability was 2.5-times higher than that of NTO-500 (Figure 2b). The radicals produced during the reaction, which are known to play a significant role in photodegradation, were studied by the ESR spectra [40]. Spin-trapping ESR for the generated superoxide radicals (·O_2_^−^) and hydroxyl radicals (·OH) by 5,5-dimethyl-1-pyrroline N-oxide (DMPO) trapping was carried out. As shown in Figure 2c, all the samples exhibited four strong 1:1:1:1 signals assigned to DMPO-·O_2_^−^ after visible-light irradiation for 5 min, verifying that the ·O_2_^−^ radicals were efficiently produced. The peak intensity of NPTO-T was higher than that of NTO-500. NPTO-600 presented the highest intensity. This was further confirmed by the ·OH-related ESR (Figure 2d). All the samples exhibited four strong 1:2:2:1 signals assigned to DMPO-·OH after visible-light irradiation for 5 min, identifying that the ·OH radicals can also be produced in these samples under irradiation. In addition, the charge-separation-related photocurrent densities (Figure S5b) were consistent with the results of photocatalytic activities and radical-related ESR.

A contradiction between the depressed photocatalytic performance and optimized N doping content of NPTO-650 (atomic content 4.22%, as shown in Table S1) may be attributed to the associated defects (deep-energy-level O_v_ and Ti^3+^ defects; Figure 1f) during the nitridation process. Those defects act as impurity energy levels under the conduction band to trap the photogenerated electrons and weaken the O_2_ reduction ability [17]. Accordingly, eliminating the associated defects is expected to improve the photocatalytic performance of NPTO-650, since the N-rich doping greatly improved the visible-light absorption (Figure 1c).

### 3.2. Defect Healing

It is generally accepted that the O_v_ and Ti^3+^ defects can be healed by the oxidation process. For comparison, a common calcination process under an O_2_ atmosphere at 400 °C was used to treat NPTO-650. The optimized R_O_2__-NPTO-650 showed extended light absorption (Figure S6a,c) and high photocatalytic activity for acetaldehyde degradation (Figure S6b,d). However, the doped N easily escapes at such a high temperature. A more effective and simpler photo-Fenton-treated reaction was therefore performed. Based on the DRS analysis (Figure S7a), the visible-light absorption of NPTO-650 was gradually eliminated with the prolonging of the immersion time in the H_2_O_2_ solution at room temperature. The optimized R_H_2_O_2__-NPTO-650 was prepared. It showed better light absorption (Figure S7a) and photocatalytic activity than those of R_O_2__-NPTO-650 (Figure S7b). Interestingly, this healing process can be improved by irradiating with a Xe lamp. The optimized sample R_L-H_2_O_2__-NPTO-650 showed much higher activity than R_H_2_O_2__-NPTO-650. As compared to R_H_2_O_2__-NPTO-650, R_L-H_2_O_2__-NPTO-650 showed better results, as shown in Figure S8.

**Figure 3:**
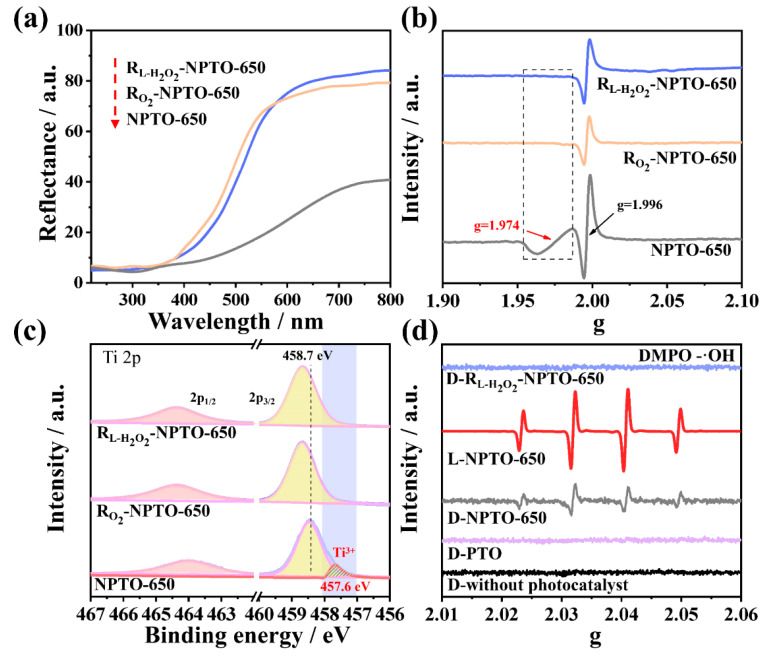
(**a**) DRS, (**b**) ESR spectra, and (**c**) Ti 2*p* XPS of NPTO-650, R_O_2__-NPTO-650, and R_L-H_2_O_2__-NPTO-650. (**d**) DMPO spin-trapping ESR spectra of hydroxyl radicals over different samples under dark (D-) or visible light (L-).

According to the XRD (Figure S9a) and Raman spectra (Figure S9b), there was barely a difference between the samples healed by different strategies. The DRS of NPTO, R_O_2__-NPTO-650, and R_L-H_2_O_2__-NPTO-650 (Figure 3a) showed that the Ti^3+^ defect-related visible-light absorption of NPTO-650 was greatly reduced. When compared to R_O_2__-NPTO-650, the absorption band edge of R_L-H_2_O_2__-NPTO-650 was slightly red-shifted to 600 nm, implying more doped N retaining in R_L-H_2_O_2__-NPTO-650. The HAADF-STEM image and corresponding EDS mapping images of R_L-H_2_O_2__-NPTO-650 showed that the N and P elements were uniformly dispersed (Figure S9c). It was further confirmed by N 1*s* XPS analysis (Figure S10a) that both R_O_2__-NPTO-650 and R_L-H_2_O_2__-NPTO-650 were unchanged in the interstitial nitrogen (399.6 eV), but had a significant decrease in the substituted nitrogen (395.6 eV) as compared to NPTO-650. The content of substituted nitrogen in R_L-H_2_O_2__-NPTO-650 was slightly higher than that in R_O_2__-NPTO-650. The XPS valence band spectrum (Figure S10b) revealed the valence band of NPTO-650 to be 1.7 eV. However, those of R_O_2__-NPTO-650 and R_L-H_2_O_2__-NPTO-650 were shifted to 2.2 eV and 2.0 eV, respectively. Figure S10c shows that the bandgap of NPTO-650 was 1.74 eV. However, those of R_O_2__-NPTO-650 and R_L-H_2_O_2__-NPTO-650 were shifted to 2.32 eV and 2.15 eV, respectively. ESR (Figure 3b) clearly showed the disappearance of the signal of Ti^3+^ defects at g = 1.974 in both R_O_2__-NPTO-650 and R_L-H_2_O_2__-NPTO-650. The signal at g = 1.996, which belonged to the defect of R_L-H_2_O_2__-NPTO-650, was higher than R_O_2__-NPTO-650. In addition, Ti 2*p* XPS (Figure 3c) showed that Ti^3+^ can be effectively healed.

DMPO spin-trapping ESR spectra were applied to verify the photo-Fenton-treated healing strategy. In Figure 3d, it is not possible to detect any signal in the system with the presence of H_2_O_2_ and DMPO, even with additional PTO samples in the dark. However, the NPTO-650 induced H_2_O_2_ reduction to produce ·OH, which was trapped by the DMPO in dark (D-NPTO-650) to generate DMPO-·OH with a weak signal. This signal was dramatically enhanced under visible-light irradiation (L-NPTO-650). However, R_L-H_2_O_2__-NPTO-650 cannot induce the H_2_O_2_ reduction reaction under the dark condition (D-R_L-H_2_O_2__-NPTO-650), which may be attributed to the absence of Ti^3+^, as confirmed in Figure 3b,c. Accordingly, this smart photo-Fenton-treated healing strategy can be described as the following equation: H_2_O_2_ + Ti^3+^ = Ti^4+^ + OH^−^ + ·OH.

## 4. Discussion

**Figure 4:**
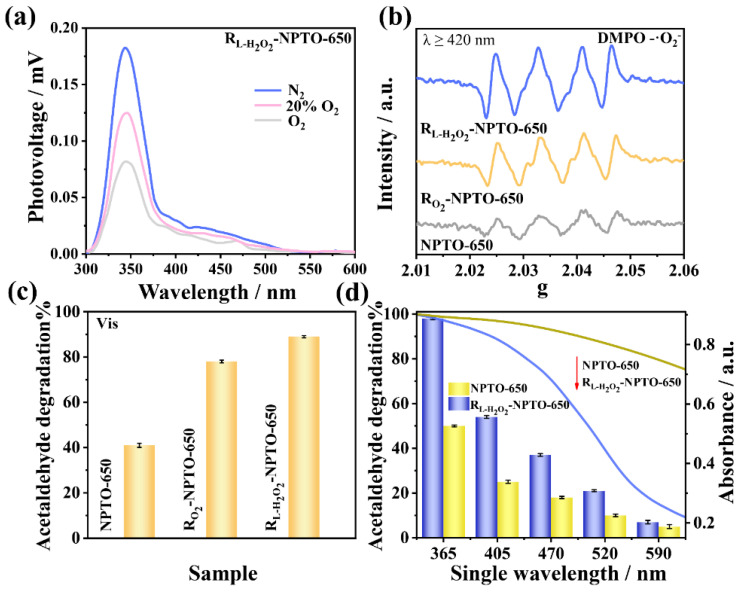
(**a**) AC-SPS responses of R_L-H_2_O_2__-NPTO-650 in different atmospheres. (**b**) DMPO spin-trapping ESR of superoxide radicals under visible-light irradiation of NPTO-650, R_O_2__-NPTO-650, and R_L-H_2_O_2__-NPTO-650. (**c**) Photocatalytic acetaldehyde degradation performance of NPTO-650, R_O_2__-NPTO-650, and R_L-H_2_O_2__-NPTO-650 under visible light. (**d**) Photocatalytic acetaldehyde degradation performance under LED light with different single wavelengths and UV-Vis absorption spectra of R_L-H_2_O_2__-NPTO-650 and NPTO-650.

In order to clarify the mechanism of charge separation in the optimized sample R_L-H_2_O_2__-NPTO-650, atmosphere-controlled surface photovoltage spectra (AC-SPS) were measured. As shown in Figure 4a, R_L-H_2_O_2__-NPTO-650 displayed the highest response under a N_2_ atmosphere, which decreased with the increasing content of O_2_ in the atmosphere. Because the doped N species in R_L-H_2_O_2__-NPTO-650 can capture the photogenerated holes, the photogenerated electrons have an easier time diffusing to the surface of the material, which resulted in the SPS response [13,33]. Moreover, the SPS results were further supported by the photoelectrochemical measurement (Figure S11a).

Based on the ESR results, both ·O_2_ (Figure 4b) and ·OH (Figure S11b) production from R_L-H_2_O_2__-NPTO-650 were the highest among all samples, implying the most enhanced charge separation. Photocatalytic acetaldehyde degradation under different light irradiation was measured. As shown in Figure 4c, the R_L-H_2_O_2__-NPTO-650 sample displayed the highest activity, which was twice as high as the NPTO-650 sample under visible-light irradiation for 1 h. Meanwhile, the R_L-H_2_O_2__-NPTO-650 sample exhibited a good stability for acetaldehyde degradation (Figure S11c). The activities of R_L-H_2_O_2__-NPTO-650 and NPTO-650 under LED light with different single wavelength irradiations were investigated (Figure 4d). One can see that the trend of single-wavelength irradiating activity matches well with the optical absorption of the photocatalysts, suggesting that the activity was indeed induced by the excitation of the sample. The R_L-H_2_O_2__-NPTO-650 exhibited activities when irradiated with light from the ultraviolet to visible regions. Even under 590 nm single-wavelength-light irradiation, it also displayed obvious activity. Acetaldehyde can be almost completely degraded after 15 min under UV-visible-light irradiation over R_L-H_2_O_2__-NPTO-650, as shown in Figure S11d, which is better than the commercial P25 TiO_2_. As shown in Figure S12a, the rate constant *k* of the samples was in the order of R_L-H_2_O_2__-NPTO-650 > R_H_2_O_2__-NPTO-650 > R_O_2__-NPTO-650 > NPTO-650. Compared with previous work, our sample showed very good activity, as shown Table S2. The in suit FT-IR spectra were carried out to study the mechanism. Both in dark and light irradiation, the peaks of the intermediates during the degradation of acetaldehyde were observed in R_L-H_2_O_2__-NPTO-650, indicating a more efficient adsorption and degradation capacity (Figure S12b). Besides, the photo-Fenton-treated healing strategy was also used for the treatment of NPTO-600. DRS (Figure S13a) indicated the bandgap of R_L-H_2_O_2__-NPTO-650 to be smaller than R_L-H_2_O_2__-NPTO-600 due to the N-rich doping. There was no obvious difference in the defect-related light absorption between them. Accordingly, the photocatalytic activity of NPTO-600 can also be improved after the healing process, but it was still lower than that of R_L-H_2_O_2__-NPTO-650 (Figure S13b).

**Figure 5:**
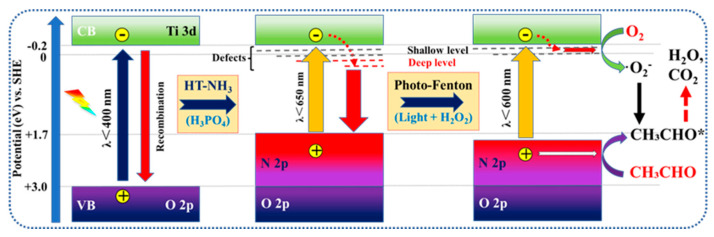
Mechanism of defect-engineering-related charge separation and photocatalytic reaction process on R_L-H_2_O_2__-NPTO-650.

Based on the above discussion, a detailed mechanism is illustrated in Figure 5. Thanks to the high thermal stability of H_3_PO_4_-modified anatase TiO_2_, N-rich doped TiO_2_ with an excellent light absorption property was successfully prepared. However, the associated deep-energy-level defects (Ti^3+^ and partial O_v_) were also formed along with the high-temperature nitridation process, which acted as the photogenerated electron acceptor and was adverse to O_2_ activation. As for this, the associated deep-energy-level defects were effectively healed, but appropriately doped N species and shallow-energy-level defects were retained by the smart photo-Fenton-treated healing strategy. After that, doped N species and shallow-energy-level defects can capture the photogenerated h^+^ and e^−^, respectively, to enhance charge separation and promote the subsequent reaction, so as to perform a good photocatalytic activity of acetaldehyde degradation.

## 5. Conclusions

In summary, N-rich doped anatase TiO_2_ was successfully fabricated by high-temperature nitridation under a NH_3_ atmosphere, depending on the H_3_PO_4_-modified TiO_2_. The doped N was demonstrated to extend visible-light absorption and trap photogenerated h^+^ to enhance charge separation. The associated deep-energy-evel defects (O_v_ and Ti^3+^) were successfully healed by a smart photo-Fenton-treated healing strategy to improve O_2_ activation and enhance charge separation. The visible-light activity for gas-phase acetaldehyde degradation of the healed N-doped TiO_2_ was twice as high as the untreated one. It also displayed better activity as compared to P25 TiO_2_ under UV-visible-light irradiation. This study sheds light on the importance of N-rich doping and modulating the associated defects for developing high-efficiency photocatalysts.

## Data Availability

Not applicable.

## Supplementary Materials

The following Supporting Information can be downloaded at: https://www.mdpi.com/article/10.3390/nano12091564/s1, Table S1: Nitrogen atomic content of NTO-500 and NPTO-T samples; Table S2: Comparison of our work with other previous work about N-doped-TiO_2_-based photocatalysts for acetaldehyde degradation; Figure S1: The power spectrum vs. wavelength of the Xe lamp and LED lamp; Figures S2–S4: Structural characterization of TO and PTO; Figure S5: (a) XPS valence band spectra of TO, PTO, NPTO-600, and NPTO-650. (b) Photocurrent densities of NTO-500 and different NPTO-Ts under visible-light irradiation; Figure S6: DRS and photocatalytic acetaldehyde degradation performance of R_O_2__-t-T-NPTO-650; Figure S7: DRS and photocatalytic acetaldehyde degradation performance of R_H_2_O_2__-t-NPTO-650 and R_L-H_2_O_2__-t-NPTO-650; Figure S8: ESR spectra, (b) DMPO spin-trapping ESR spectra of superoxide radicals under visible-light irradiation; Figure S9: (a) XRD patterns and (b) Raman spectra of NPTO-650, R_O_2__-NPTO-650, R_H_2_O_2__-NPTO-650, and R_L-H_2_O_2__-NPTO-650. (c) TEM image with corresponding EDX mapping of R_L-H_2_O_2__-NPTO-650; Figure S10: N 1*s* XPS (a), XPS valence band spectra (b), and bandgap (c) of NPTO-650, R_O_2__-NPTO-650, R_H_2_O_2__-NPTO-650, and R_L-H_2_O_2__-NPTO-650; Figure S11: (a) Current densities of NPTO-650, R_O_2__-NPTO-650, R_H_2_O_2__-NPTO-650, and R_L-H_2_O_2__-NPTO-650 under visible-light irradiation. (b) DMPO spin-trapping ESR spectra for hydroxyl radicals of NPTO-650, R_O_2__-NPTO-650, R_H_2_O_2__-NPTO-650, and R_L-H_2_O_2__-NPTO-650 under visible-light irradiation. (c) Cycling tests of R_L-H_2_O_2__-NPTO-650 under visible-light irradiation. (d) Photocatalytic acetaldehyde degradation performance of NPTO-650, R_O_2__-NPTO-650, R_H_2_O_2__-NPTO-650, R_L-H_2_O_2__-NPTO-650, and P25 under UV-Vis light irradiation; Figure S12: (a) Pseudo-first-order decay curves of NPTO-650, R_O_2__-NPTO-650, R_H_2_O_2__-NPTO-650, and R_L-H_2_O_2__-NPTO-650 for acetaldehyde degradation of the under visible-light irradiation. (b) In situ FT-IR spectra of NPTO-600 and R_L-H_2_O_2__-NPTO-650 for photocatalytic acetaldehyde degradation under visible-light irradiation for 1 h; Figure S13: (a) DRS of R_L-H_2_O_2__-NPTO-600 and R_L-H_2_O_2__-NPTO-650. (b) Photocatalytic acetaldehyde degradation performance of NPTO-600 and NPTO-650 before and after photo-Fenton-treated healing process. References [41,42,43] are cited in the Supplementary Materials.
