# Supplementary material for: N-Rich Doped Anatase TiO2 with Smart Defect Engineering as Efficient Photocatalysts for Acetaldehyde Degradation
Source: Nanomaterials (Basel). 2022 May 5;12(9):1564. doi: 10.3390/nano12091564 (PMC9105496; doi:10.3390/nano12091564)
Supplement: Supplementary file 1 [file nanomaterials-12-01564-s001.zip › nanomaterials-1693445-supplementary.pdf]

Support Information

N-Rich Doped Anatase TiO_2 with Smart Defect Engineering as Efficient Photocatalysts for Acetaldehyde Degradation

Mingzhuo Wei, Zhijun Li *, Peijiao Chen, Lei Sun, Shilin Kang, Tianwei Dou, Yang Qu * and Liqiang Jing *

Key Laboratory of Functional Inorganic Materials Chemistry (Ministry of Education), School of Chemistry and Materials Science, International Joint Research Center for Catalytic Technology, Heilongjiang University, Harbin 150080, China; mingzhuo0828@163.com (M.W.); chenpeijiao0@163.com (P.C.); 2021022@hlju.edu.cn (L.S.); 18846423501@163.com (S.K.); doutw1992@126.com (T.D.)

* Correspondence: 2018011@hlju.edu.cn (Z.L.); quyang@hlju.edu.cn (Y.Q.); jinglq@hlju.edu.cn (L.J.)

Experimental Section

1.1. Synthesis of anatase TiO_2 nanoparticles

In this experiment, a mixed solution of 5 mL of tetrabutyl titanate and 5 mL of absolute ethanol was added dropwise to a mixture of 5 mL of absolute ethanol, 1 mL of ultrapure water, and 1 mL of nitric acid. Stirring was continued for 30 min. The mixed solution was transferred to a 100 mL autoclave, and hydrothermally heated at 160 ^{o}C for 6 h. After cooling to room temperature, the obtained sol product was washed with double distilled water and absolute ethanol for 6 times. After drying, the sample was roasted in a muffle furnace at 450 °C for 2 h (5 °C /min) to obtain anatase nanoparticles. The sample was named TO.

1.2. DMPO spin-trapping ESR measurement

1.2.1. Hydroxyl radical measurement

·OH species were confirmed by electron paramagnetic resonance (ESR) which were carried out on a Bruker EMXPLUS model spectrometer operating at the X-band frequency. The reactive ·OH species were detected with the assist of 5,5-dimethyl-1-pyrroline N-oxide (DMPO) as a spin trap reagent under visible-light irradiation. The concentration of DMPO was 50 mM and ·OH species were determined in aqueous phase.

1.2.2. Superoxide radical measurement

$\cdot O_2^-$ species were confirmed by ESR which were carried out on a Bruker EMX plus model spectrometer operating at the X-band frequency. The reactive $\cdot O_2^-$ species were detected with the assist of DMPO as a spin trap under visible-light irradiation. The concentration of DMPO was 50 mM and $\cdot O_2^-$ were determined in methanol phase.

1.3. Photoelectrochemical and electrochemical measurements

The film electrode was fabricated as follows: firstly, 10 mg of samples, 0.1 mL of Nafion and 0.9 mL of ethanol were mixed to the slurry thoroughly. Then the slurry was coated on the FTO glass electrode (1.0 cm × 1.0 cm). At last, the coated electrode was dried at 60 °C for 30 min. Photoelectrochemical (PEC) and electrochemical (EC) measurements were carried out on the IVIUM V13806 electrochemical workstation with traditional three-electrode system. The as-prepared sample films were used as working electrodes in a sealed quartz cell. A platinum plate (99.9%) and saturated KCl Ag/AgCl electrode were used as the counter electrode and reference electrode, respectively. 0.2 M $NaSO_4$ solution was used as electrolyte. High-purity N_2 or O_2 (99.999%) were employed to bubble through the electrolyte to keep the saturated gas in the EC experiment. PEC experiments were performed in a quartz cell using a 300 W xenon lamp with a cut-off filter ($\lambda > 420$ nm) as the illumination source. All the experiments were performed at room temperature (about 25 ± 3 °C).

1.4. In situ Fourier transform infrared (FT-IR) spectroscopy

In situ FT-IR spectroscopic experiments were performed on a Nicolet iS50 FT-IR spectrometer equipped with a MCT-A detector cooled with liquid nitrogen. The catalyst powder (150–200 mg) was pressed into a self-supported wafer. Before measurement, the catalysts were compressed and held in a custom-fabricated IR reaction chamber which was sealed with CaF_2 windows and pretreated in high-purity nitrogen gas (99.999%) flow (30 mL min^{-1}) at 100 °C for 2 h to remove the impurity adsorbed on the surface. The samples were subsequently cooled down to room temperature and the background spectrum was collected. After that, gas mixture (800 ppm acetaldehyde, 20% O_2, and about 80% N_2) was introduced into the system, and the FT-IR spectrum was collected after the adsorption was saturated. The samples were then purged with nitrogen to remove the free and physical adsorbed acetaldehyde, and then FT-IR spectra were collected. Finally, the infrared spectrum after light irradiation for 1 h was collected. The temperature and gas flow were monitored using an on-site transient analysis platform (CRCP-7070-B, Xianquan).

Table S1. Nitrogen atomic content of NTO-500 and NPTO-T samples.

Sample	N<br>Atomic Content (%)
NTO-500	0.54
NPTO-500	0.62
NPTO-550	2.41
NPTO-600	3.12
NPTO-650	4.22

Table S2. Comparison of our work with other previous work about N doped TiO_2-based photocatalysts for acetaldehyde degradation.

Materials	Degradation percentage (%)	K min^{-1}	Illuminant	References
$R_{L-H_2O_2}$-NPTO-650	89	0.03371	150W xenon lamp	This work
N-TiO_2	60	0.01896	80W mercury lamp	[1]
N-TiO_2	41	0.00936	80W mercury lamp	[2]
N_{CQDs}-TiO_2	57	0.00174	150W xenon lamp	[3]

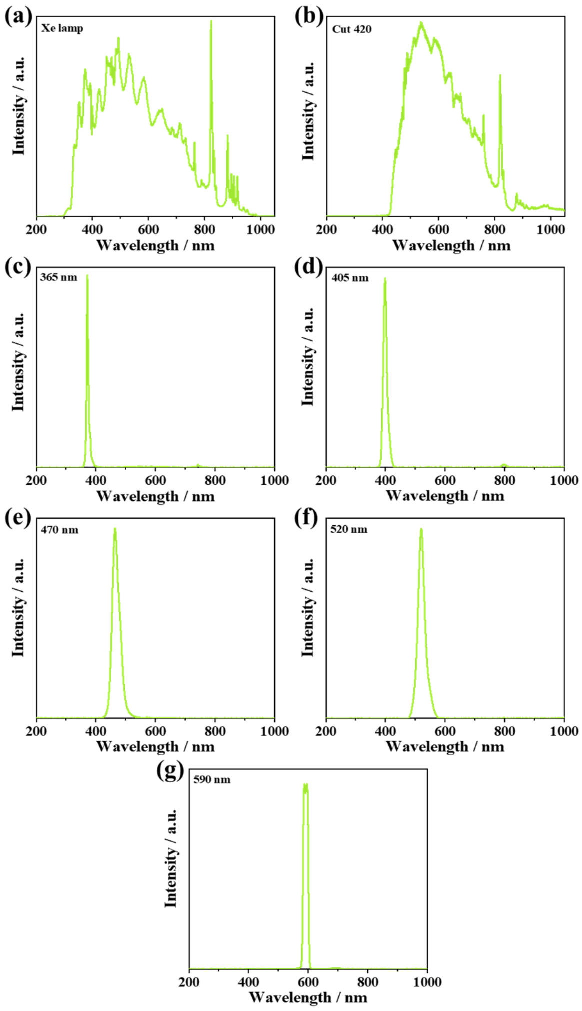

Figure S1. Power spectrum vs. wavelength of Xe lamp (**a**), 420 cut-off of Xe lamp (**b**) and LED light with different single wavelengths (**c**–**g**).

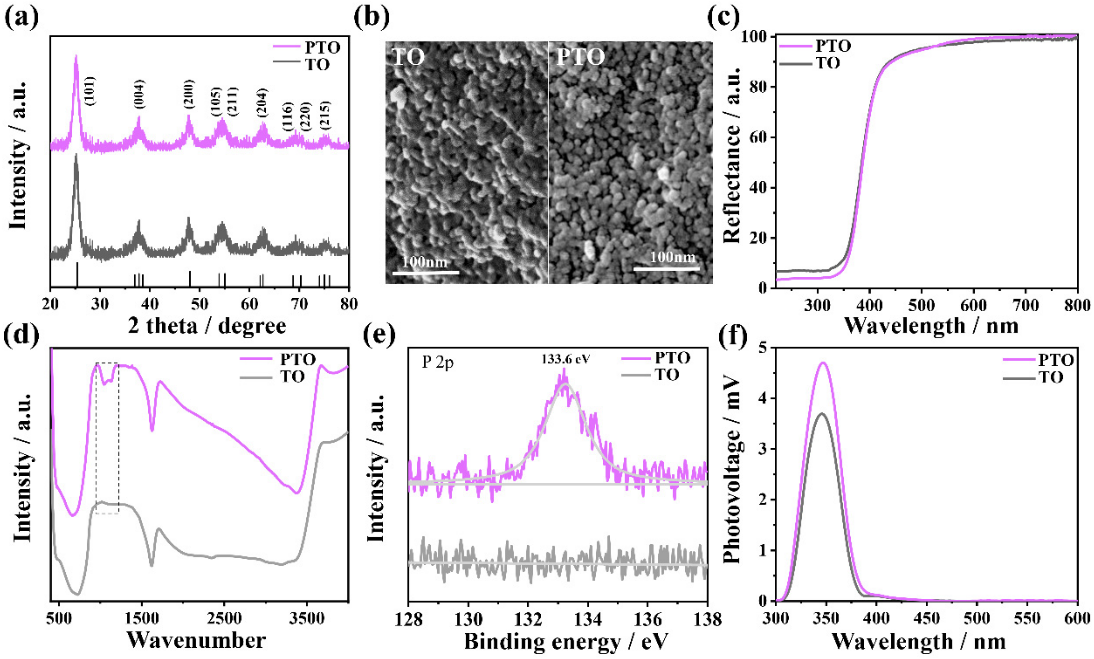

Figure S2. XRD patterns (**a**), SEM images (**b**), DRS (**c**), FT-IR spectra (**d**), P 2*p* XPS (**e**) and SPS responses (**f**) of TO and PTO.

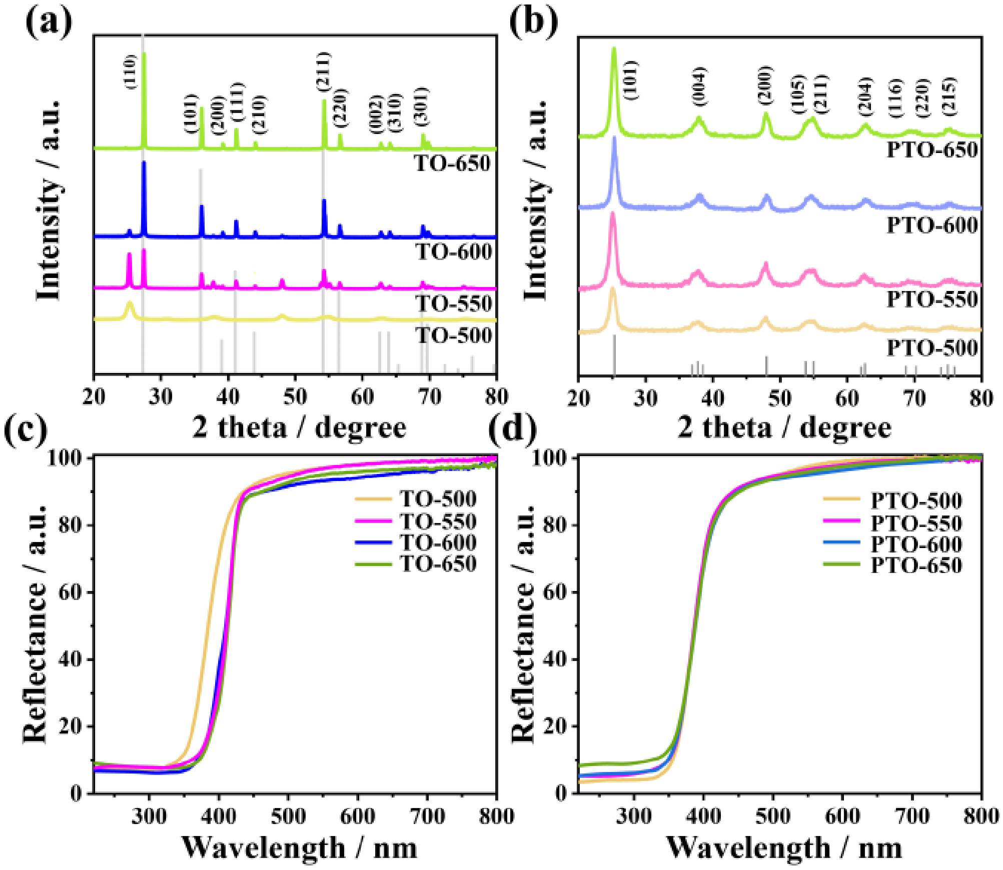

Figure S3. XRD patterns of TO (**a**) and PTO (**b**) with different calcination temperature. DRS of TO (**c**) and PTO (**d**) with different calcination temperature.

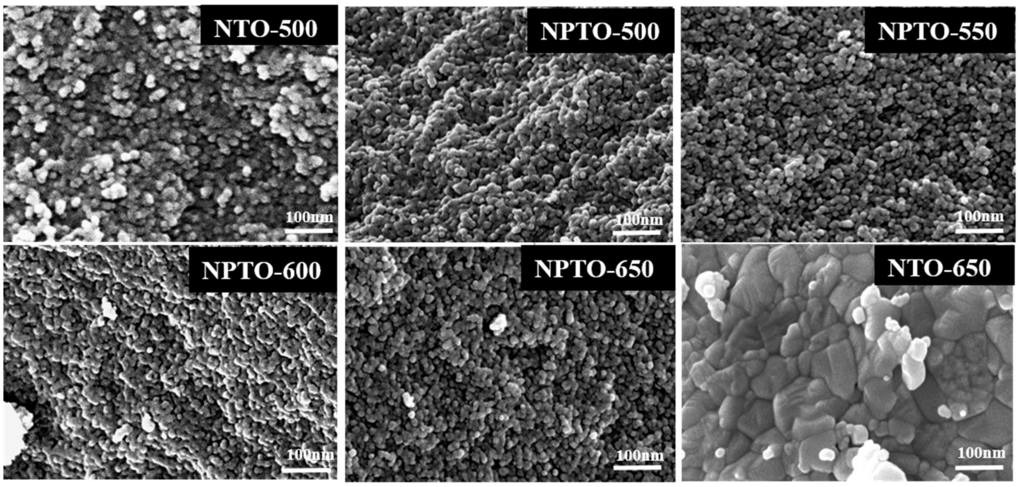

Figure S4. SEM images of NTO-500, NPTO-T and NTO-650.

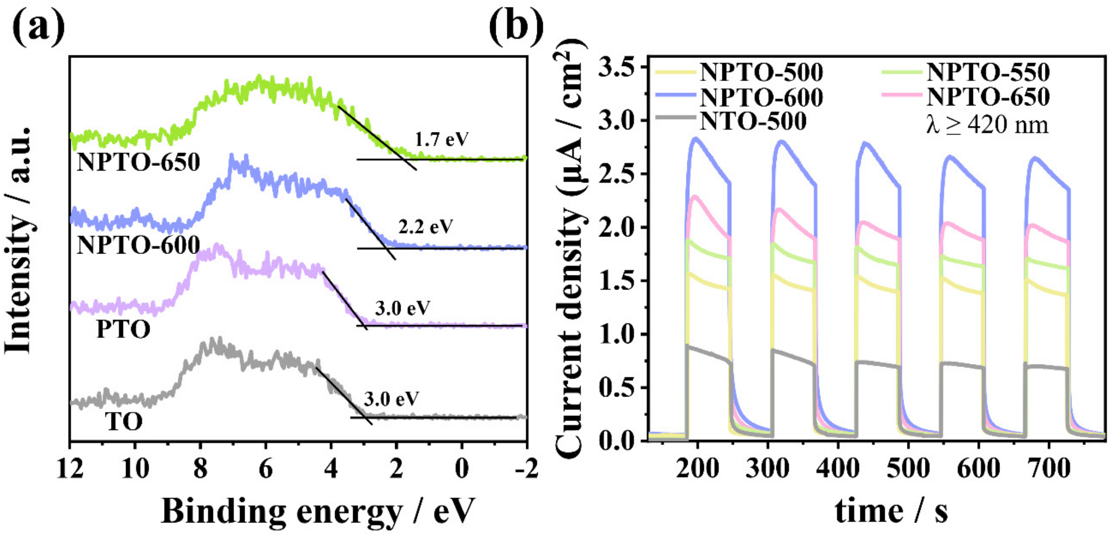

Figure S5. (**a**) XPS valence band spectra of TO, PTO, NPTO-600 and NPTO-650. (**b**) Photocurrent densities of NTO-500 and different NPTO-T under visible-light irradiation.

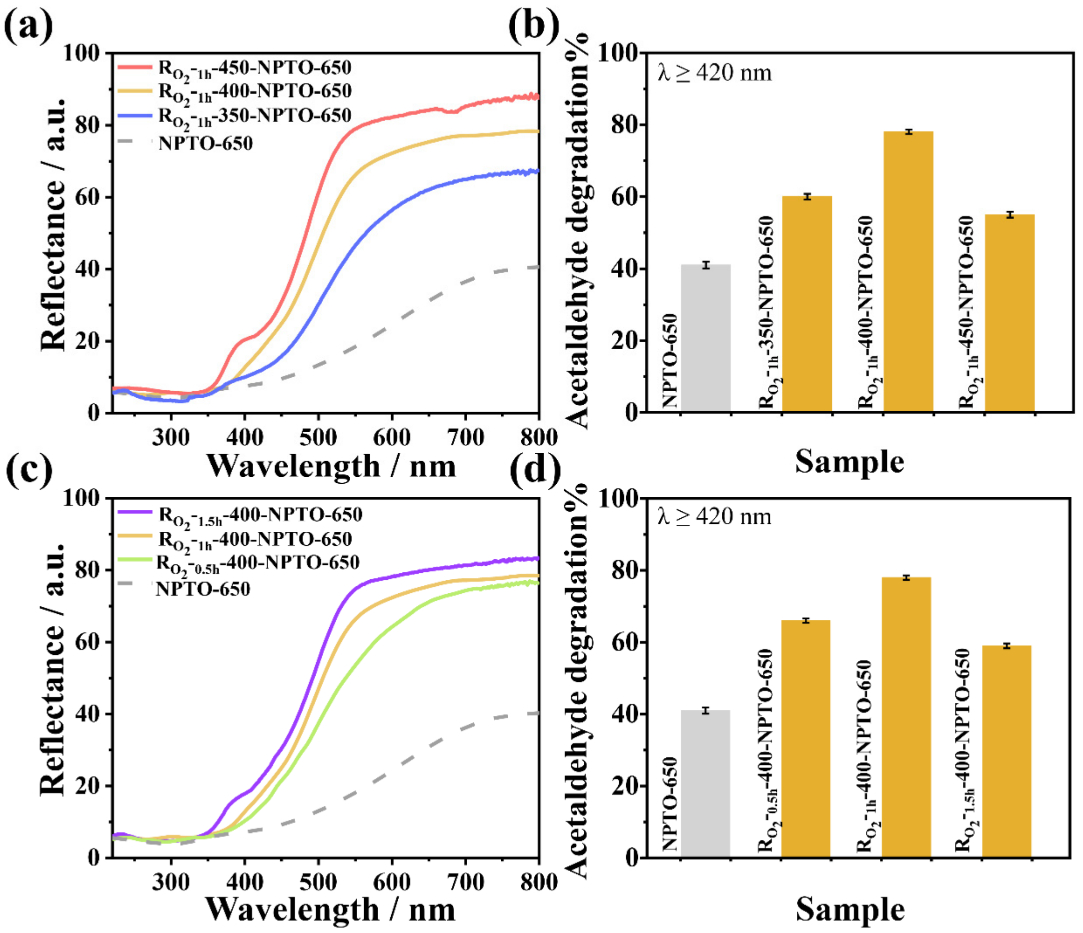

Figure S6. (**a**) DRS and (**b**) Photocatalytic acetaldehyde degradation performance of R_{O_2}-1h-T-NPTO-650 under visible-light irradiation for 1 h ("T" is the treatment temperature under O_2 atmosphere, T = 350, 400 and 450 °C, respectively). (**c**) DRS and (**d**) Photocatalytic acetaldehyde degradation performance of R_{O_2}-t-400-NPTO-650 under visible-light irradiation at 400 °C ("t" is the time of thermal treatment under O_2 atmosphere, t = 0.5, 1, and 1.5 h).

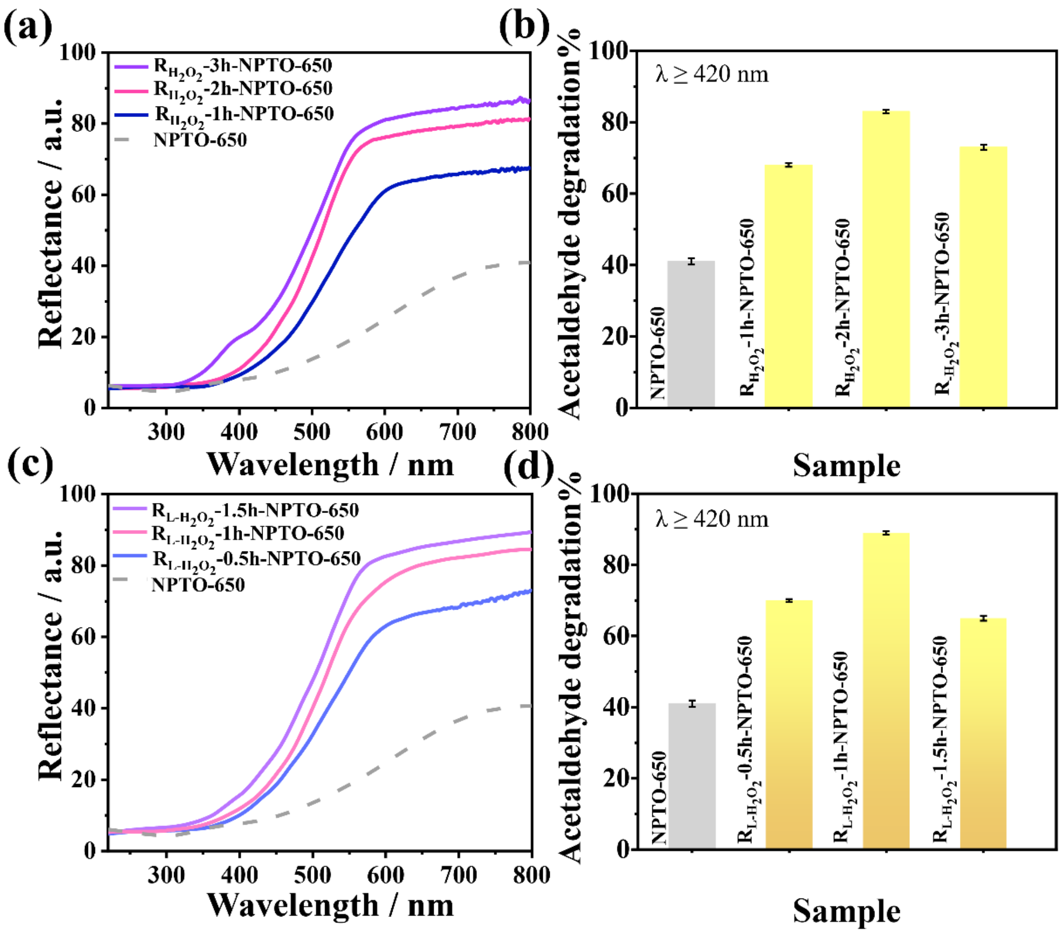

Figure S7. (**a**) DRS and (**b**) Photocatalytic acetaldehyde degradation performance of $R_{H_2O_2}$-t-NPTO-650 under visible-light irradiation for 1 h ("t" is the dipping time of NPTO-650 in the H_2O_2 solution at room temperature, t = 1, 2, and 3 h, respectively.) (**c**) DRS and (**d**) Photocatalytic acetaldehyde degradation performance of $R_{L-H_2O_2}$-t-NPTO-650 under visible-light irradiation for 1 h ("t" is the dipping time of NPTO-650 in the H_2O_2 solution under visible-light irradiation at room temperature, t = 0.5, 1, and 1.5 h).

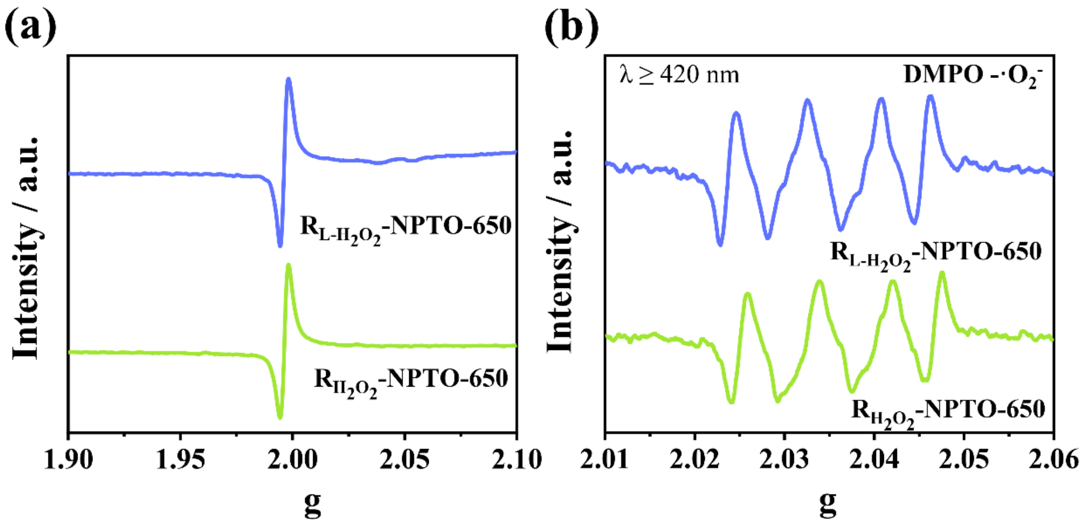

Figure S8. (**a**) ESR spectra, (**b**) DMPO spin-trapping ESR spectra of superoxide radicals under visible-light irradiation and.

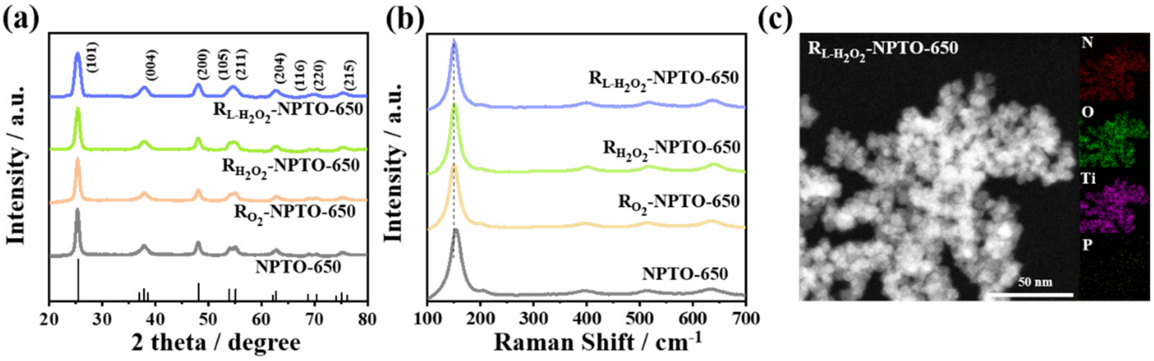

Figure S9. (**a**) XRD patterns and (**b**) Raman spectra of NPTO-650, R_{O_2}-NPTO-650, $R_{H_2O_2}$-NPTO-650 and $R_{L-H_2O_2}$-NPTO-650. (**c**) TEM image with corresponding EDX mapping of $R_{L-H_2O_2}$-NPTO-650.

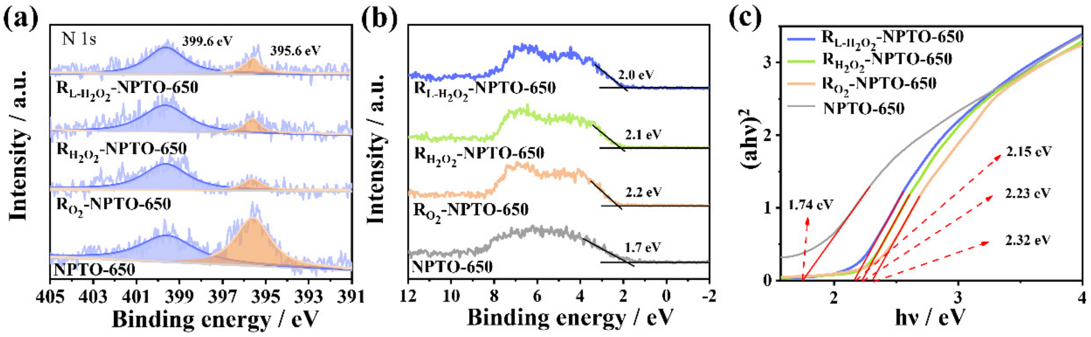

Figure S10. N 1*s* XPS (**a**), XPS valence band spectra (**b**), and bandgap evaluation (**c**) of NPTO-650, R_{O_2}-NPTO-650, $R_{H_2O_2}$-NPTO-650 and $R_{L-H_2O_2}$-NPTO-650.

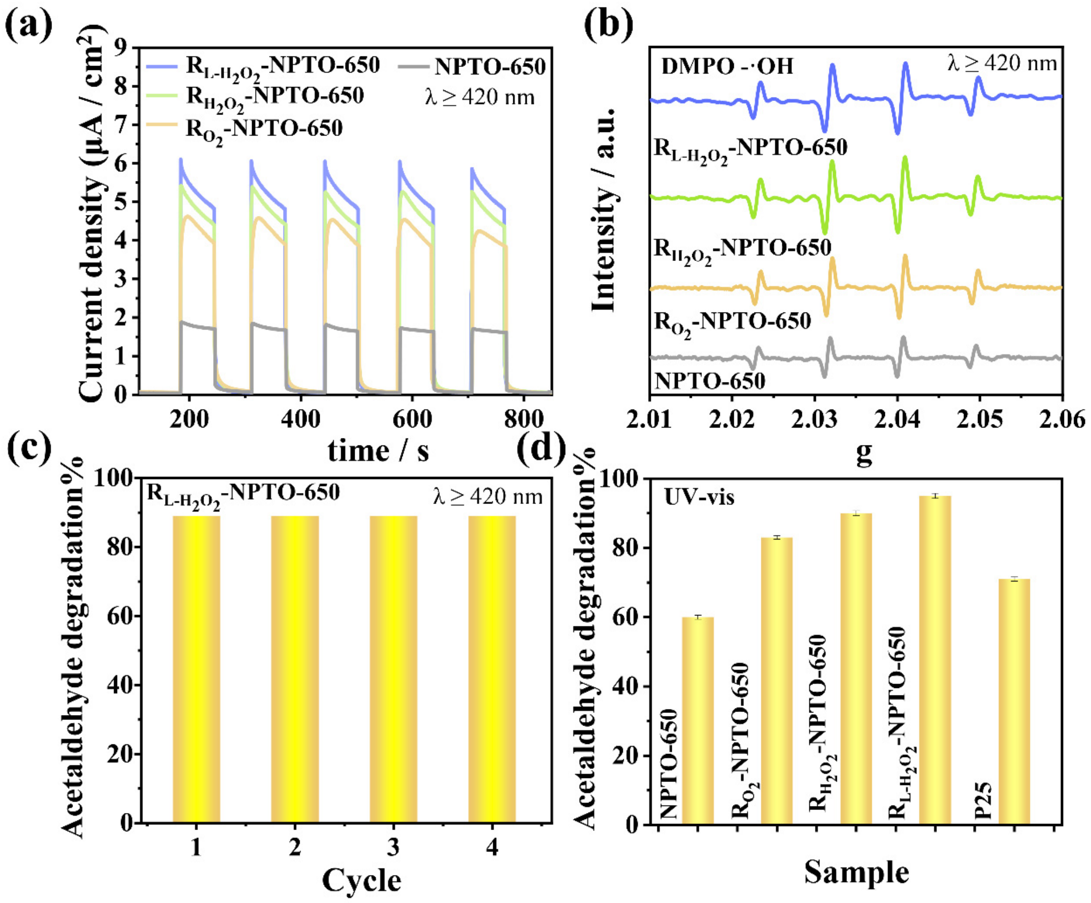

Figure S11. (**a**) Current densities of NPTO-650, R_{O_2}-NPTO-650, $R_{H_2O_2}$-NPTO-650 and $R_{L-H_2O_2}$-NPTO-650 under visible-light irradiation. (**b**) DMPO spin-trapping ESR spectra for hydroxyl radicals of NPTO-650, R_{O_2}-NPTO-650, $R_{H_2O_2}$-NPTO-650 and $R_{L-H_2O_2}$-NPTO-650 under visible-light irradiation. (**c**) Cycling tests of $R_{L-H_2O_2}$-NPTO-650 under visible-light irradiation. (**d**) Photocatalytic acetaldehyde degradation of NPTO-650, RO2-NPTO-650, $R_{H_2O_2}$-NPTO-650, $R_{L-H_2O_2}$-NPTO-650 and P25 under UV-vis light irradidation.

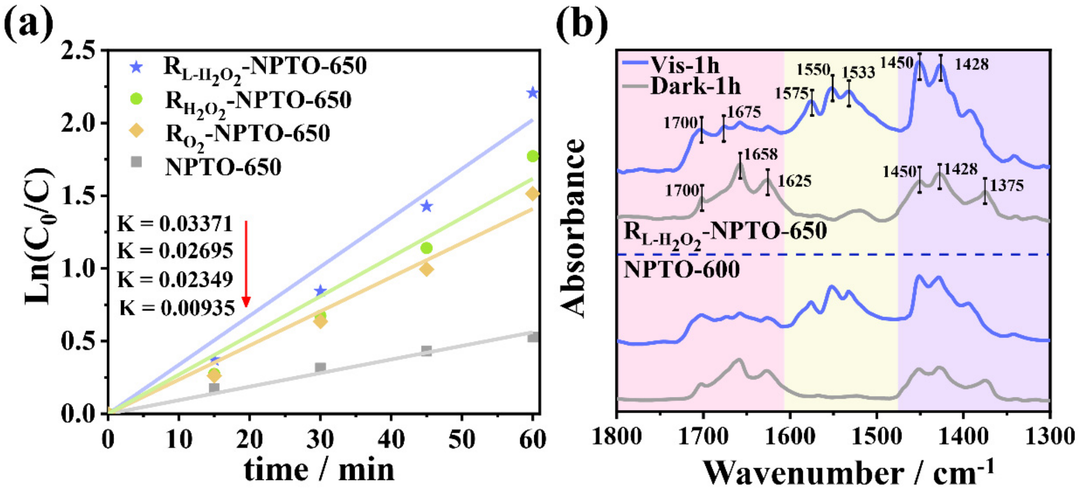

Figure S12. (**a**) Pseudo-first-order decay curves of NPTO-650, R_{O_2}-NPTO-650, $R_{H_2O_2}$-NPTO-650 and $R_{L-H_2O_2}$-NPTO-650 for acetaldehyde degradation of the under visible-light irradiation. (**b**) Insitu FT-IR specrtra of NPTO-600 and $R_{L-H_2O_2}$-NPTO-650 for photocatalytic acetaldehyde degradation under visible-light irradiation for 1 h.

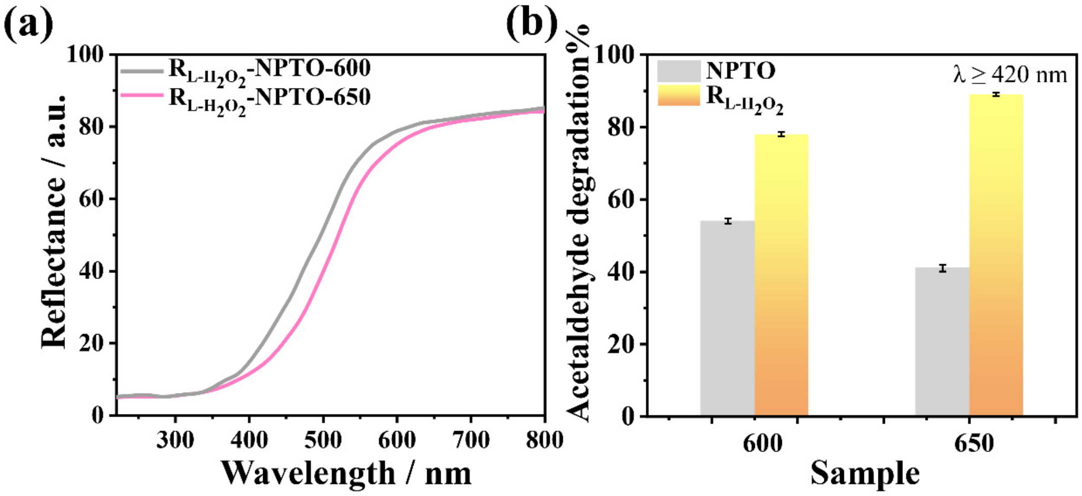

Figure S13. (**a**) DRS of $R_{L-H_2O_2}$-NPTO-600 and $R_{L-H_2O_2}$-NPTO-650. (**b**) Photocatalytic acetaldehyde degradation performance of NPTO-600 and NPTO-650 before and after photo-Fenton like repairing process.

References

1. Khalilzadeh, A.; Fatemi, S. Modification of Nano-TiO_2 by Doping with Nitrogen and Fluorine and Study Acetaldehyde Removal under Visible Light Irradiation. *Clean Technol. Envir.* **2013**, *16*, 629–636.
2. Vahidzadeh, E.; Fatemi, S.; Nouralishahi, A. Synthesis of a Nitrogen-Doped Titanium Dioxide–Reduced Graphene Oxide Nano-composite for Photocatalysis under Visible Light Irradiation. *Particuology* **2018**, *41*, 48–57.
3. Xie, C.; Fan, T.; Wang, A.; Chen, S. L. Enhanced Visible-Light Photocatalytic Activity of a TiO_2 Membrane-Assisted with N-Doped Carbon Quantum Dots and SiO_2 Opal Photonic Crystal. *Ind. Eng. Chem. Res.* **2018**, *58*, 120–127.
